# Optometrists’ perceptions of vision impairment services in public hospitals of Limpopo province

**DOI:** 10.4102/ajod.v14i0.1559

**Published:** 2025-06-03

**Authors:** Modjadji M. Leshabane, Nishanee Rampersad, Khathutshelo P. Mashige

**Affiliations:** 1 Department of Optometry, College of Health Sciences, University of KwaZulu-Natal, Durban, South Africa

**Keywords:** vision impairment, vision impairment services, rehabilitation, assistive devices, low vision, blindness

## Abstract

**Background:**

Vision impairment (VI) services aim to mitigate the effect of VI and provide opportunities for visually impaired individuals to actively participate in their daily activities.

**Objectives:**

To determine optometrists’ perceptions regarding VI services in public hospitals within Limpopo province, South Africa.

**Method:**

A descriptive, quantitative, cross-sectional study was conducted between January and August 2023 across 37 public hospitals, using a structured questionnaire. Data obtained from the participants’ responses were analysed to describe the level of VI services.

**Results:**

The study sample included 65 optometrists with 71% female, yielding a response rate of 83%. Over 90% of the participants were aware of the World Health Organization definition of VI. The majority of participants (54%) reported referring patients with VI to a hospital multidisciplinary team, while less than 50% provided optimal spectacle correction. The main barriers to providing VI services were: the lack of assistive devices (97%), and equipment (95%), poor access (80%), insufficient space (66%), and the lack of training (66%). The primary barriers to the uptake of VI services were the lack of awareness (86%) and the cost of VI services (80%).

**Conclusion:**

The provision of VI services in Limpopo province is currently limited. The factors contributing to the limited VI services are avoidable; therefore, efforts to enhance the availability of equipment, access and provision of comprehensive VI services are crucial to improving the quality of life for affected individuals utilising public hospitals in Limpopo province.

**Contribution:**

The study describes the optometrists’ perceptions of VI services in public hospitals.

## Introduction

Vision impairment (VI) is defined as a functional limitation of the eye/s or visual system because of a disorder, which can result in visual disability or visual handicap (Heath, Kishiki & Courtright 2007; World Health Organization [WHO] 2019a). Vision impairment includes low vision (visual acuity [VA] less than 6/18 to 3/60) and blindness (VA worse than 3/60 to light perception) based on presenting VA (WHO 2022). Vision impairment can manifest as reduced VA or contrast sensitivity, visual field loss, photophobia, colour vision loss, diplopia, visual distortion, visual perceptual difficulties or any combination of the abovementioned symptoms (Heath et al. 2007). An individual with functional low vision has impairment of visual functioning even after treatment and/or standard refractive correction, with VA less than 6/18 to light perception or visual field less than 10 degree from the point of fixation, but uses or is potentially able to use vision for the planning and/or execution of a task (WHO 2008).

Globally, approximately 295 million individuals have moderate to severe VI, 43 million are blind and 510 have near VI (Bourne et al. 2021a). Furthermore, about 90% of the visually impaired individuals live in low- and middle-income countries (LMICs) (Ackland, Resnikoff & Bourne 2017; Bourne et al. 2021a). In South Africa, VI accounted for 9.9% among all disabilities, making it the largest disability group in the country (Statistics South Africa 2024). The main global causes of VI include uncorrected refractive errors (UREs), cataracts, diabetic retinopathy and age-related macular degeneration (WHO 2021). In South Africa, a recent study identified UREs, cataracts and glaucoma as the most common causes of VI (Xulu-Kasaba & Kalinda 2022).

Vision impairment restricts affected individuals from attaining optimum function and independence in their daily lives, leading to decreased quality of life and contributing to poor psycho-social well-being, physical health, economic participation and educational achievements (Bassey, Ellison & Walker 2019; Bourne et al. 2021a; Watermeyer, et al. 2024; WHO 2021). To improve their daily functioning, individuals with VI require comprehensive VI services encompassing promotional, preventative, treatment and/or rehabilitation services. People with irreversible VI (congenital or acquired) require low vision care and rehabilitation services (WHO 2019a). Several studies have shown that comprehensive VI services are effective in improving functioning for activities of daily living (ADL) and psychological well-being in affected individuals (Da Silva et al. 2014; McKnight, Crudden & McDonnall 2021; Ovenseri-Ogbomo et al. 2016).

Low vision care and rehabilitation services include vision assessment and goal identification, refraction, provision of assistive devices and training on their use, psychological counselling on the underlying condition, adaptation and use of residual vision, mobility and orientation training, occupational rehabilitation and environmental modification, referral to special education and job placement services (Monye, Kyari & Momoh 2020; Owsley et al. 2009; WHO 2019a). The services require a professional multidisciplinary approach which involves personnel including optometrists, ophthalmologists, ophthalmic nurses, occupational therapists, orientation and mobility trainers, psychologists, community–based rehabilitation workers, audiologists, social workers, special educators, physiotherapists and low vision therapists to ensure comprehensive rehabilitation services (Heath et al. 2007; Oduntan 2008; WHO 2017).

Optometry is defined as a profession concerned with the eyes and related structures, as well as vision, visual systems and vision information processing in humans (Bergin 2017). Optometrists are licensed or registered primary healthcare practitioners of the eye and visual system who provide comprehensive eye and vision care, which includes refraction and dispensing, detecting and/or diagnosis and management of eye disease and the rehabilitation of the condition of the visual system (Health Professional Council of South Africa [HPCSA] 2025; World Council of Optometrists 2025). Given the scope of practice of optometrists, they possess the requisite skills and expertise to deliver services related to VI (Naidoo et al. 2023).

Globally, the demand for VI services is projected to rise because of the ageing population, prevailing lifestyle comorbidities and complications arising from non-communicable systemic and/or ocular diseases (Bourne et al. 2021a; WHO 2017). The WHO action plan prioritises reducing avoidable VI as a global public health issue and ensuring access to rehabilitation services for individuals with irreversible VI. This empowerment aims to enable full participation in social, economic, political and cultural aspects of life (WHO 2013). Despite these efforts, significant inequalities and gaps persist in the awareness, access and uptake of VI services worldwide. Approximately 5% of the population with chronic VI has access to low vision care and rehabilitation services worldwide (Chiang et al. 2011). In most instances, LMICs are underserved or the services are inadequate and generally poor (Bourne et al. 2021b; WHO 2017, 2021).

In South Africa, provision of low vision and rehabilitation services is variable, inadequate and gravely constrained in most rural parts of the country (Oduntan 2007; Sacharowitz 2005; Watermeyer et al. 2024). The low vision care and rehabilitation services are mainly offered by the four optometric teaching institutions, one college, few public special schools, several non-profit organisations (NPOs) and few private practice optometrists (Oduntan 2007; Sacharowitz 2005). Barriers to the provision, access and uptake of VI services are multifaceted, involving healthcare system constraints, individual factors, societal issues; and they vary across and within countries (Bourne et al. 2021a, 2021b; Chiang et al. 2011; Wallace et al. 2020).

The Limpopo province is the fifth most populous province in South Africa, with a predominantly rural landscape (Limpopo Provincial Government 2020; Statistics South Africa 2019). While numerous studies have examined the epidemiology of VI in various parts of Limpopo province (Maake & Oduntan 2015; Mabaso & Oduntan 2014; Oduntan et al. 2003), there is a notable paucity of literature addressing the awareness, availability and barriers to accessing VI services in the public hospitals of Limpopo province. This study aimed to elucidate the perceptions of optometrists regarding VI services in the public hospitals of Limpopo province. The findings are anticipated to be instrumental for policymakers, eye care personnel and the Department of Health in facilitating informed planning, resource allocation and management of VI, ultimately enhancing the quality of life of affected individuals and their families.

## Research methods and design

### Study design

The study used a descriptive, quantitative, cross-sectional design to explore optometrists’ perceptions of VI services in the public hospitals of Limpopo province.

### Study site and population

The study was conducted in public hospitals providing optometry services in Limpopo province, South Africa. During the study period (January–September 2023), 37 public hospitals employing 81 optometrists who offered eye care services in the province. The optometry services offered across these hospitals were relatively homogeneous concerning patient assessment, diagnosis and disease management.

### Sampling strategy

Convenience sampling was used to recruit participants from all public hospitals within the province. A total of 81 optometrists employed in public hospitals were recruited to participate in the study. Three of these optometrists participated in the pilot study. Consequently, a saturated sample of the remaining 78 optometrists was included in the study. The questionnaire was distributed electronically to these 78 optometrists, and 65 optometrists completed and returned the questionnaire.

### Data collection

A modified, validated structured questionnaire was used for data collection. The design of the questionnaire was informed by a comprehensive review of previous literature (Jose et al. 2016; Kyeremeh & Mashige 2018). A pilot study was conducted with three optometrists, not included in the main sample, and two academic optometrists to evaluate content validity, suitability of the questionnaire and the data collection procedures. Based on feedback from the pilot study, five questions were deleted and four were rephrased to reduce ambiguity. The results from the pilot study were excluded from the final data analysis. The final questionnaire comprised 36 close-ended questions divided into five sections: demographic information, awareness, availability, barriers to the provision and uptake of VI services. The questionnaire was disseminated to participants via Google Forms. The Google Form also included the study information, and participants provided consent to participate in the study before accessing the questionnaire. To enhance the response rate, the researcher sent a follow-up email 2 weeks after the initial distribution, and made calls to participants 1 week later to remind them of the study and the completion of the questionnaire. This approach was deemed necessary to maximise the response rate, as surveys are typically constrained by low response rates (Agustini 2018; Fincham 2008).

### Data analysis

Data were collected electronically and analysed using the Statistical Package for Social Sciences (SPSS) version 29 (IBM, Chicago, Illinois, United States). The numerical and categorical data responses to the questions were analysed using descriptive statistics to determine frequencies. The chi-square test was used to compare awareness, availability and barriers to the provision of VI services based on participants’ years of work experience. A *p*-value < 0.05 was considered statistically significant.

### Ethical considerations

Approval to conduct the study was obtained from the Humanities and Social Science Research Ethics Committee (HSSREC/00004472/2022) of the University of KwaZulu-Natal. Thereafter, gatekeeper permission and approval were obtained from the Limpopo Provincial Department of Health (LP_2022-12-004). Anonymity was ensured by providing all participants with individual codes.

## Results

### Demographic characteristics

A total of 65 optometrists from 37 public hospitals completed the questionnaire, yielding a response rate of 83%. Table 1 presents the demographic information of participants. The sample predominantly comprised females (*n* = 46, 71%) and nearly all participants (*n* = 64, 98%) had a Bachelor of Optometry qualification. The majority of participants had 11 or more years of working experience (*n* = 40, 62%) and were employed at primary-level hospitals (*n* = 43, 66%). All participants were involved in providing general eye care services, whereas a limited number of participants (*n* ≤ 6) provided orthoptic vision, contact lens or low vision care services. A small number of participants (*n* = 6, 9%) reported offering low vision care services. In addition, low vision care was identified as an area of interest for approximately 30% of the sample.

**TABLE 1:** Demographic information of the participants (*N* = 65).

Demographic information	Variables	Frequency (*n*)	%
Gender	Male	19	29
	Female	46	71
Qualification	Diploma in optometry	0	0
	Bachelor of optometry	64	98
	Master of optometry	1	2
Years of work experience	0–5	0	0
	6–10	25	38
	≥ 11	40	62
Level of care	Primary	43	66
	Secondary	19	29
	Tertiary	3	5
Specialised eye care services provided	General eye care	65	100
	Orthoptic vision care	2	3
	Contact lens care	2	3
	Low vision care	6	9
Participants’ specialised field of interest	Binocular vision care	7	11
	Contact lens care	13	20
	Environmental optometry	1	2
	Low vision care	19	29
	Ocular pathology and emergency care	19 8	29 12

### Awareness of vision impairment

The average number of patients with VI examined per month ranged from 20 to 300 with a mean (standard deviation) of 106.92 (± 91.223). Table 2 illustrates participants’ awareness of VI stratified based on years of working experience. While over 90% of the sample were aware of the WHO definition of VI, only 50% of the participants used the WHO criteria to classify a person with VI. There was an almost equal distribution of participants who classified a person with VI based on poor vision in both eyes (*n* = 16, 25%), and those who based it on patient needs (*n* = 15, 23%).

**TABLE 2:** Awareness of vision impairment services based on years of working experience (*N* = 65).

Questions	*N*		≥ 11 years		6–10 years		*p*-value
	*n*	%	*n*	%	*n*	%	
**Are you aware of the WHO definition of VI?**	-	-	-	-	-	-	0.312
Yes	59	92	36	90	23	92	-
No	1	2	0	-	1	4	-
Not sure	5	8	4	10	1	4	-
**In your clinic, you classify a person with VI based on:**	-	-	-	-	-	-	0.134
Patient needs (e.g., unable to perform daily activities and/or hobbies)	15	23	11	28	4	16	-
Poor vision in both eyes	16	25	12	30	4	16	-
WHO criteria	34	52	17	42	17	68	-
**You classify a person with low vision when the VA in the better eye is worse than:**	-	-	-	-	-	-	0.032
1/60 to light perception	2	3	2	5	0	0	-
3/60 to light perception	4	6	2	5	2	8	-
6/18 but equal to or better than 3/60	43	66	31	78	12	48	-
6/36 but equal to or better than 3/60	6	9	1	3	5	20	-
6/60 but equal to or better than 3/60	10	15	4	10	6	24	-
**You classify a person with low vision when the VF from the point of fixation is worse than:**	-	-	-	-	-	-	0.222
10^o^	22	34	13	33	9	36	-
20^o^	24	37	12	30	12	48	-
30^o^	13	20	11	28	2	8	-
Not sure	6	9	4	10	2	8	-
**You classify a person with blindness when the VA in the better eye is worse than:**	-	-	-	-	-	-	0.017
1/60 to no light perception	18	28	15	38	3	12	-
3/60 to no light perception	15	23	10	25	5	20	-
6/18 but equal to or better than 3/60	5	8	4	10	1	4	-
6/36 but equal to or better than 3/60	2	3	0	0	2	8	-
6/60 but equal to or better than 3/60	23	35	9	23	14	56	-
Not sure	2	3	2	5	0	0	-
**You classify a person with blindness when the VF from the point of fixation is worse than:**	-	-	-	-	-	-	0.515
10^o^	49	75	29	72	20	80	-
20^o^	10	15	6	15	4	16	-
30^o^	6	9	5	13	1	4	-
**What is vision rehabilitation?**	-	-	-	-	-	-	-
Training to use low vision devices	53	82	31	78	22	88	0.537
Mobility and orientation training	50	77	30	75	20	80	0.897
Adaptive training for job	32	49	21	53	11	44	0.756
Counselling	39	60	25	63	14	56	0.824

VI, vision impairment; WHO, World Health Organization; VF, visual field; VA, visual acuity.

In terms of classifying VI to include individuals who might benefit on vision rehabilitation services, the majority of participants classified the person with low vision when the VA in the better eye was worse than 6/18 but equal to or better than 3/60 (*n* = 43, 66%). Fewer than 50% of all participants classified a person with low vision when the visual field (VF) was worse than 20^o^ from the point of fixation. Participants with more than 11 years of working experience (78%) had a high percentage of classifying a person with low vision when the VA in the better eye was worse than 6/18 but equal to or better than 3/60, compared with those with less than 10 years of working experience (48%). Less than 30% (*n* = 15) of the participants classified a person with blindness when the VA in the better eye was worse than 3/60 to no light perception. In contrast, more than 70% of the participants classified a person with blindness when the VF was worse than 10^o^ from the point of fixation. Irrespective of years of working experience, 25% or less participants classified an individual with blindness when the VA in the better eye was worse than 3/60.

The majority of participants identified vision rehabilitation as encompassing training on the use of low vision devices (82%), training for mobility and orientation (77%), counselling (60%) and adaptive training for employment (49%). There was no association between participants’ years of working experience and their awareness of the WHO definition of VI, criteria to classify a person with VI, low vision and blindness based on the VF from the point of fixation and participants’ awareness of vision rehabilitation services (*p* > 0.05). However, an association was found between participants’ years of working experience and classifying an individual with low vision when the VA in the better eye was worse than 6/18 and blindness when the VA in the better eye was worse than 3/60 (*p* < 0.05).

### Availability of vision impairment services

Table 3 presents the participants’ responses regarding the availability of VI services in their local areas as stratified based on their years of working experience. The majority of participants (54%) reported referring patients with VI to a hospital multi-disciplinary team. Less than 50% of participants provided optimal spectacle correction, some form of vision rehabilitation services or referred patients to low vision care centres. Most participants engaged with ophthalmologists (92%), psychologists (78%) and occupational therapists (71%) when providing VI services. Nearly 50% of the participants indicated the absence of a referral centre for irreversible VI services in their districts. Only eight participants frequently referred patients with irreversible VI to the centre for vision rehabilitation services, while the majority (*n* = 57, 88%) either never or rarely referred patients.

**TABLE 3:** Availability of vision impairment services based on years of working experience (*N* = 65).

Questions	*N*		≥ 11 years		6–10 years		*p*-value
	*n*	%	*n*	%	*n*	%	
**What do you do when you get a patient with VI?**							
Refer to the hospital MDT	35	54	20	50	15	60	0.431
Provide the best possible spectacle correction	29	45	19	48	10	40	0.554
Provide rehabilitation services	20	31	13	33	7	28	0.702
Refer to low vision care centre	17	26	8	20	9	36	0.842
**Which healthcare professionals do you collaborate with in VI care services?**							
Ophthalmologist	60	92	37	93	23	92	0.929
Ophthalmic nurses	25	38	15	38	10	40	0.968
Occupational therapist	46	71	27	68	19	76	0.763
Psychologist	51	78	30	75	21	84	0.679
General practitioner	18	28	11	28	7	28	0.983
Social worker	33	51	21	53	12	48	0.905
**What type of referral centre provides vision rehabilitation services in your area?**	-	-	-	-	-	-	0.157
Hospital low vision clinic	12	18	11	28	1	2	-
Hospital MDT	1	2	1	3	0	0	-
Non-profit organisation	15	23	9	23	6	24	-
Special school	3	5	3	8	0	0	-
None	34	52	17	43	17	68	-
**How often do you refer to the centre that provides vision rehabilitation services?**	-	-	-	-	-	-	0.791
Often	8	12	4	10	4	16	-
Rare	17	26	10	25	7	25	-
Very rare	12	18	7	18	5	20	-
Never	28	43	20	50	8	32	-
**What are the barriers/challenges for referral to a centre that provides vision rehabilitation services?**	-	-	-	-	-	-	-
Lack of contacts	25	38	15	38	10	40	0.968
Lack of referral procedure	62	95	38	95	24	96	0.852
Not sure	12	18	9	23	3	12	0.544
Access	5	8	3	8	2	8	0.981
**If you refer, do you receive any feedback from the referral centre regarding your patients?**	-	-	-	-	-	-	0.982
Yes	13	20	8	20	5	20	-
No	52	80	32	80	20	80	-

VI, vision impairment; MDT, multi-disciplinary team.

The lack of a referral procedure (95%) was identified as a major barrier to referring patients to these centres. No statistically significant association was found between participants’ years of working experience and collaboration with other health care professionals in managing patients with VI, the type of service provided to patients with VI, the referral of patients to centres offering vision rehabilitation care services or the challenges encountered in referring patients to such centres (*p* > 0.05).

### Barriers to the provision and uptake of vision impairment services

**FIGURE 1:**
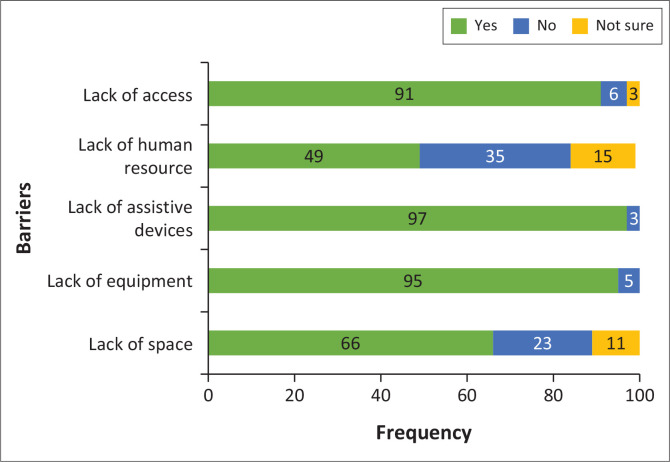
Barriers for vision impairment services inherent in the health care system.

**FIGURE 2:**
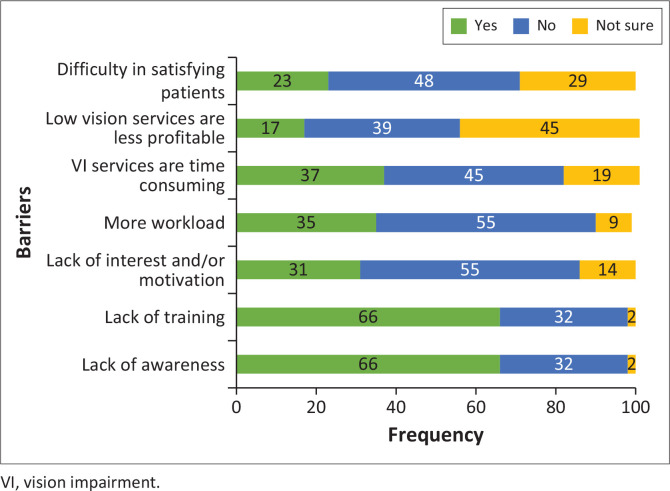
Barriers for practitioners in providing vision impairment services.

**FIGURE 3:**
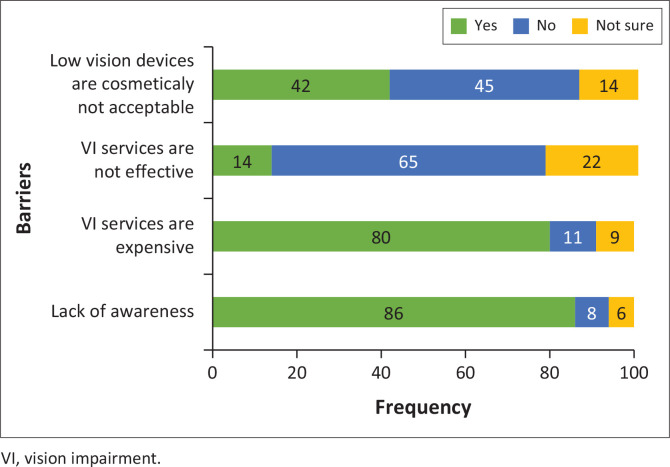
Barriers for the uptake of vision impairment services.

Figure 1 to Figure 3 illustrate barriers in the provision of VI services within the health care system, barriers faced by practitioners and barriers encountered by patients in accessing VI services, respectively. In terms of barriers inherent within the health care system, the primary obstacles identified included the lack of assistive devices (97%), the lack of equipment (95%), the lack of access (80%) and insufficient space (66%) (Figure 1). Although 55% of participants expressed interest in providing VI services (Figure 2), more than 60% of participants reported a lack of training and awareness while only 35% of participants reported increased workload as barriers faced by practitioners in the provision of VI services. Despite perceiving VI services as effective (65%), participants reported the lack of awareness (86%), and the cost of VI services (80%) as main barriers to their uptake of these services in the province (Figure 3).

## Discussion

Vision impairment interferes with developmental growth in children and significantly impacts the quality of life among the adult population (Bassey et al. 2019; Bourne et al. 2021a; WHO 2021). The demand for VI services is anticipated to increase globally because of ageing population and prevailing lifestyle comorbidities (Bourne et al. 2021a; WHO 2021). This study aimed to describe optometrists’ perceptions of VI services in public hospitals in the Limpopo province, South Africa.

The findings from this study on awareness of VI showed that 90% of the participants were aware of the WHO definition of VI. However, only 50% of the participants applied the WHO criteria to identify and classify individuals with VI. This discrepancy in VI classifications may stem from the lack of standardised guidelines for VI classifications or the absence of necessary enablers for implementation, such as diagnostic equipment in public hospitals. Interestingly, the majority of those who adhered and used the WHO criteria in classifying an individual as having VI were those with 10 years or less of working experience (68%). This trend may be attributed to their greater involvement in patient care and a higher likelihood of engaging in continuous education and skill development than their more experienced counterparts. Inconsistencies in the classification of VI contribute to inaccurate estimations of the number of individuals who could benefit from VI services, including refractive error correction, cataract surgery, low vision care and taking into account the person’s vision-related problems and needs, and vision rehabilitation (Dijk, Kishiki & Courtright 2014). This misclassification may therefore lead to affected individuals being denied access to essential services as accurate estimates of VI are crucial for planning effective eye care services and monitoring progress (WHO 2019a). The use of the International Classification of Diseases (ICD) for classifying VI, as recommended by the WHO, is commonly employed in clinical settings and research studies (Ali et al. 2022; Bourne et al. 2021b; Seid et al. 2022; WHO 2019b).

Vision impairment services aim to optimise the use of residual vision through the use of assistive devices, medical and surgical interventions, psychological counselling and environmental adaptations (WHO 2019a). Early reports indicated that the main causes of VI in parts of Limpopo Province were correctable and/or preventable (Maake & Oduntan 2015; Mabaso & Oduntan 2014; Magakwe, Xulu-Kasaba & Hansraj 2020; Oduntan et al. 2003). However, findings from this study revealed that only 9% of the participants offered low vision care services, less than 50% of participants provided optimal spectacle correction and approximately 54% referred patients with VI to ophthalmologists, psychologists or occupational therapists. Only eight participants (12%) frequently referred patients for vision rehabilitation services, while the majority (88%) either never or rarely made such referrals.

Despite that refractive error correction services are cost-effective and feasible to implement (WHO 2021), poor provision of spectacles and other visual assistive devices might be because of anecdotal reports suggesting insufficient budget for optical devices and poor procurement processes at public hospitals in Limpopo province. While similar findings of inadequate refractive error coverage services were reported in Saudi Arabia (Ovenseri-Ogbomo & Alghamdi 2021) and Zambia (Kapatamoyo et al. 2023), the budget and procurement constraints could further worsen the provision and access to low vision care at public hospitals because of the expensive costs of these services, thus leaving the majority of the people who rely on public eye health care services underserved.

Almost all hospitals in Limpopo province had a significant cataract backlog and only three public hospitals were offering cataract surgery services at the time of study. An early study in parts of Limpopo province found that patients were placed on the cataract surgery waiting lists for longer periods (Khoza et al. 2020a). It was found that insufficient ophthalmology personnel, shortage of equipment and consumables for cataract surgery services contributed to limited provision of cataract surgery services in the province (Khoza et al. 2020a, 2020b).

The increase in referrals of patients with VI to the ophthalmologists could be reduced by strengthening the co-management of eye diseases between the optometrists with ocular therapeutics privileges and ophthalmologists. Naidoo et al. (2023) showed that optometrists are best placed to contribute to the disease control strategy to reduce the global burden of VI. The lack of a vision rehabilitation plan and inadequate referrals of those who might benefit from the low vision care and rehabilitation services could be because of the lack of referral guidelines and insufficient vision rehabilitation centres in the province. Consistent with other studies, low vision care and rehabilitation services were reported to be more constrained in LMICs (Oduntan 2007; WHO 2017, 2021).

Participants identified the lack of assistive devices, equipment and access as major barriers inherent within the health care system in the provision of VI services. Assistive devices, both optical and non-optical, can restore vision and/or enhance functionality of individuals with VI, thus their provision significantly impacts the vision-related quality of life (Da Silva et al. 2014; Ovenseri-Ogbomo et al. 2016). However, similar findings were reported in other studies (Javed, Afghani & Zafar 2015; Kapatamoyo et al. 2023; Kyeremeh & Mashige 2021; Lim et al. 2014; Monye et al. 2020; Wallace et al. 2020), where the lack of equipment, access and assistive devices were cited as primary barriers to provision of VI services. The absence of adequate equipment adversely affects the quality of services offered, leading to inadequate diagnosis, inappropriate referrals and redundant skills with overburdening of the receiving institutions.

While 55% of participants expressed interest in providing VI services and 29% provided low vision care and rehabilitation services, barriers such as inadequate human resource and training, limited awareness and increased workload were identified as significant obstacles for practitioners in delivering these services. The inadequate human resource which might be the reason for increased workload could be because of shortage of optometrists in some hospitals as majority of optometrists are predominantly located in the private sector (Naidoo et al. 2023). This could be the contributing factor for poor integration of low vision care and rehabilitation services in the public sector as optometrists may mainly focus on providing refractive services and screening for diseases (Naidoo et al. 2023). The lack of awareness among practitioners may be attributed to insufficient involvement in continuous professional education and low interest in professional skill development. This deficiency in awareness has been reported as a major barrier to effective VI services (Dilkash et al. 2021; Jose et al. 2016). Furthermore, inadequate training leads to incompetent practitioners and ineffective service delivery. Continuous educational training is important to ensure that practitioners maintain up-to-date skills and developments in their fields and adherence to such training is mandatory (HPCSA 2021). These limitations and inadequacies deprive persons with VI to attain and maintain maximum independence, full physical, mental, social and vocational ability, and full inclusion and participation in all aspects of life and could infringe on their rights and well-being (Department of Social Development 2016; United Nations 2025).

Participants identified the primary barriers to the uptake of VI services as a lack of awareness and the cost of services. The lack of awareness may be attributed to the literacy levels of the population being served and poor eye-care-seeking behaviours (WHO 2021). A deficiency in awareness and knowledge about available services negatively impacts the utilisation of these services (Akuffo et al. 2020; Ntsoane et al. 2012). Although public health services in South Africa are subsidised, individuals who are not fully subsidised may still be unable to afford their portion of the hospital bill because of their socio-economic status (Bourne et al. 2021a; Limpopo Provincial Government 2020). Furthermore, inadequate coverage for refractive error correction and cataract surgery in the province may contribute to the ineffectiveness of services, resulting in a lack of patient satisfaction because of unmet expectations.

## Strength and limitations

The limitations of this study include its hospital-based design, which is subject to the inherent constraints of facility-based studies, such as limited generalisability of the findings. In addition, the study is susceptible to information bias because of the selection of participants being limited to optometrists. Despite these limitations, the study offers valuable insights for policymakers, and eye care practitioners, aiding in the effective planning of visual impairment services and serving as a foundation for further research.

## Conclusion

The availability and provision of VI services in Limpopo province are currently limited. Advocacy for the use of recommended guidelines is essential to ensure the delivery of quality eye care services. It is necessary to enhance the availability of equipment, improve awareness of VI services, develop and ensure implementation of guidelines for referral to improve access and provision of effective VI services. The factors contributing to the limited VI services are avoidable. Therefore, appropriate planning on provision of comprehensive VI services and resource allocation are necessary to reduce the burden of VI and ultimately improve the quality of life of affected individuals utilising the public hospitals in Limpopo province.
